# TGF-β-Containing Small Extracellular Vesicles From PM_2.5_-Activated Macrophages Induces Cardiotoxicity

**DOI:** 10.3389/fcvm.2022.917719

**Published:** 2022-07-08

**Authors:** Xiaoqi Hu, Mo Chen, Xue Cao, Xinyi Yuan, Fang Zhang, Wenjun Ding

**Affiliations:** ^1^Laboratory of Environment and Health, College of Life Sciences, University of Chinese Academy of Sciences, Beijing, China; ^2^Sino-Danish Center for Education and Research, Sino-Danish College, University of Chinese Academy of Sciences, Beijing, China

**Keywords:** PM_2.5_, macrophage, small extracellular vesicles (sEV), TGF-β, cardiotoxicity

## Abstract

Numerous epidemiological and experimental studies have demonstrated that the exposure to fine particulate matter (aerodynamic diameter <2.5 μm, PM_2.5_) was closely associated with cardiovascular morbidity and mortality. Our previous studies revealed that PM_2.5_ exposure induced cardiac dysfunction and fibrosis. However, the corresponding underlying mechanism remains largely unaddressed. Here, PM_2.5_-induced cardiotoxicity is presented to directly promote collagen deposition in cardiomyocytes through the transforming growth factor-β (TGF-β)-containing small extracellular vesicles (sEV). The sEV transition may play an important role in PM_2.5_-induced cardiac fibrosis. Firstly, long-term PM_2.5_ exposure can directly induce cardiac fibrosis and increase the level of serum sEV. Secondly, PM_2.5_ can directly activate macrophages and increase the release of tumor necrosis factor α (TNF-α), interleukin-6 (IL-6), and TGF-β-containing sEV. Thirdly, TGF-β-containing sEV increases the expression of α-smooth muscle actin (α-SMA), collagen I, and collagen III in mouse cardiac muscle HL-1 cells. Finally, TGF-β-containing sEV released from PM_2.5_-treated macrophages can increase collagen through the activation of the TGF-β-Smad2/3 signaling pathway in HL-1 cells from which some fibroblasts involved in cardiac fibrosis are thought to originate. These findings suggest that TGF-β-containing sEV from PM_2.5_-activated macrophages play a critical role in the process of increasing cardiac collagen content *via* activating the TGF-β-Smad2/3 signaling pathway.

## Highlights

- PM_2.5_ exposure increased the level of sEV, which can promote collagen deposition in cardiomyocytes.
- TGF-β-containing sEV from PM_2.5_-induced macrophages activated the TGF-β-Smad2/3 signaling pathway and caused cardiotoxicity.

## Introduction

Exposure to particulate matter (PM) with an aerodynamic diameter <2.5 μm (PM_2.5_) causes certain health risks and toxic effects on various tissues and systems (1). Epidemiological and experimental studies have demonstrated that long-term or high-concentration PM_2.5_ exposure can increase the morbidity and mortality of cardiovascular diseases (CVD) (2–4). PM induces many pathological processes, such as systemic inflammation and oxidative stress, and causes acute arterial vasoconstriction, endothelial dysfunction, arrhythmia, and procoagulation/thrombosis, which could aggravate the occurrence and development of CVD (2–4). Existing studies have indicated that PM_2.5_ exposure can induce ECG changes such as abnormality of heart rhythm, tachycardia, and T-wave reduction, and increase the level of inflammatory cell infiltration and fibrosis of cardiac tissue in mice (5, 6). Our previous research also found that PM_2.5_ induced cardiac dysfunction and fibrosis (7). However, the relevant molecular mechanisms of PM_2.5_-induced cardiac injury need to be elucidated.

Recently, small extracellular vesicles (sEV) have received extensive attention in intercellular communication and signaling (8). sEV are a group of extracellular vesicles (40–160 nm) containing nuclear acids, proteins, lipids, and metabolites (9), which are selectively loaded to regulate the biological functions in receptor cells (10). Multiple studies have found that sEV are involved in the regulation of pathological processes of cardiovascular diseases, including atherosclerosis, hypertension, and myocardial infarction (11–13). These findings indicate that sEV may play a key role in cardiac fibrosis after PM_2.5_ exposure.

To explore the mechanism of sEV regulating cardiac fibrosis after PM_2.5_ exposure, we firstly assessed cardiac injury in PM_2.5_ exposed mice (14). Then we confirmed the increase of fibrosis-related proteins in cardiomyocytes cocultured with serum sEV after PM_2.5_ exposure. To investigate whether sEV from macrophages and lung epithelial cells regulate the progression of cardiac fibrosis through body circulation, we examined the levels of key fibrosis-inducing mediators TNF-α, IL-6, and TGF-β in sEV with different exposure times. Our study indicated that TGF-β-enriched sEV, which were released from macrophages after PM_2.5_ exposure, could promote the process of cardiac fibrosis. The findings provide a reference for the cardiovascular-related diseases induced by ambient particulate matter exposure.

## Materials and Methods

### Experimental Animals

The 7-week-old C57BL/6J mice were purchased from Beijing Huafukang Bio-Technology Co., Ltd. After being adaptively fed for 1 week, mice were randomly assigned to the filtered air (FA) group and PM_2.5_ group. The PM_2.5_ concentration in the FA group ranges from 0 to 5 μg/m^−3^. During the whole exposure period, all the mice were fed with commercial mouse chow and distilled water, and the exposed warehouse was maintained under temperature (22 ± 2 °C) and relative humidity (40–60%) conditions with a 12 h light/dark cycle. After 4 months (July-October 2017) of exposure, blood samples and heart tissues were obtained for the following experiments. The whole animal studies were performed in accordance with the principles of laboratory animal care (NIH publication no. 85–23, revised 1985) and with approval from the University of Chinese Academy of Sciences Animal Care and Use Committee.

### PM_2.5_ Sampling and Preparation

The PM_2.5_ samples were collected from September to November 2016 by a medium-volume air particle sampler (TH-150D-I, Wuhan Tianhong, China) located on Zhongguancun East Road, Beijing. The PM_2.5_ particles were collected by Teflon-coated filters (diameter = 90 mm, Whatman, St. Louis, MO) and stored at −20 °C until use. To extract the PM_2.5_ sample from Teflon filters, a sonicator (Catalog No. KQ-700 V, Shumei, China) was applied for 30 min. The extracted PM_2.5_ was diluted to a final concentration of 10 mg/ml and stored at −80 °C prior to the study.

### Cell Culture and PM_2.5_ Exposure

RAW264.7, MLE-12, and HL-1 cells were grown in high-glucose Dulbecco's modified Eagle medium with 10% fetal bovine serum (FSP500, ExCell Bio, China) and 1% penicillin and streptomycin. The cells were kept in a humidified incubator at 37 °C with 5% CO_2_. RAW264.7 and MLE-12 cells were seeded into 6-well plates and exposed to 50 μg/ml PM_2.5_ for 1, 12, 24, and 48 h for total RNA extracted. After a 24-h-treatment with 50 μg/ml PM_2.5_, a conditioned medium of RAW264.7 and MLE12 cells were harvested to isolate sEV.

### Coculture

The coculture of RAW264.7 and HL-1 cells was applied in a transwell model (Corning, USA), which separates two chambers by a membrane with 0.4 μm pores. RAW264.7 cells were incubated in the upper chamber, while the HL-1 cells were cultured in the bottom chamber. RAW264.7 and HL-1 cells were cultured separately in the upper and bottom chambers until cells were grown to 80% confluence. GW4869 (Sigma-Aldrich, USA, 10 μM) was added to pre-treat RAW264.7 cells for 2 h to block sEV release. After 2 h of treatment, the upper chamber medium was removed. Subsequently, the upper chamber was inserted into the bottom chamber and exposed to 50 μg/ml of PM_2.5_ for 24 h. We extracted the cardiomyocyte protein for Western blot.

### RNA Isolation and Quantitative Real-Time PCR

Total RNA was extracted from tissues and cell lysates by using TRIzol Reagent (Invitrogen, Carlsbad, CA), and reverse transcription was performed with PrimeScript™ RT Master Mix (Takara Bio, Shiga, Japan) according to the manufacturer's instructions. Real-time PCR was performed using SYBR Premix Ex TaqII (Takara Bio, Japan) on a QuantStudio 7 Flex system (Thermo Fisher Scientific, USA). The primers used in this study are listed in Table 1. To determine the relative expression of mRNA in response to various stimuli, 18 s was used as the internal reference. The gene expression was quantified using 2^−ΔΔCt^ method.

**Table 1:** Primers used for real-time PCR.

**Primer**	**Forward primer (5^**′**^-3^**′**^)**	**Reverse primer (5^**′**^-3^**′**^)**
*α-sma*	GTCCCAGACATCAGGGAGTAA	TCGGATACTTCAGCGTCAGGA
*Mmp9*	CTGGACAGCCAGACACTAAAG	CTCGCGGCAAGTCTTCAGAG
*Col1α1*	GCTCCTCTTAGGGGCCACT	CCACGTCTCACCATTGGGG
*Col1α2*	GTAACTTCGTGCCTAGCAACA	CCTTTGTCAGAATACTGAGCAGC
*Col3α1*	CTGTAACATGGAAACTGGGGAAA	CCATAGCTGAACTGAAAACCACC
*TGF-β*	CTCCCGTGGCTTCTAGTGC	GCCTTAGTTTGGACAGGATCTG
*18s*	TTCTGGCCAACGGTCTAGACAAC	CCAGTGGTCTTGGTGTGCTGA

### Western Blot Assay

Total proteins from cardiac tissue, myocardial cell, and sEV were lysed with RIPA buffer (Beyotime, China) with 1% PMSF and protease and phosphatase inhibitor cocktails (Bimake, USA). Protein concentration was measured by enhanced BCA protein assay kit (Beyotime, China). Protein samples were analyzed on 10% sodium dodecyl sulfate polyacrylamide gel electrophoresis (SDS-PAGE) with color prestained protein marker (LABLEAD, China) and transferred to PVDF membranes (Millipore, USA). The PVDF membranes were blocked with 5% non-fat milk for 1 h at room temperature. The membranes were incubated with primary antibodies overnight at 4 °C and then incubated with diluted secondary antibody at room temperature for 1 h. Immunoreactive bands were detected with ECL reagents (Bio-Rad, CA). β-Tubulin was used as a protein-loading control. Primary antibodies F4/80 (#70076T) and E-cadherin (#3195S) were purchased from Cell Signaling Technology (CST, USA), CD63 (#ab217345), CD9 (#ab92726), α-SMA (#ab124964), and Collagen I (#ab260043) were purchased from Abcam (UK); TGF-β (#orb11468) and Collagen III (#orb371960) were purchased from Biorbyt (UK); Alix (#sc-53540) was purchased from Santa Cruz (USA); TSG101 (#28283-1-AP) was purchased from Proteintech (China); smad2 (#ET1604-22), p-smad2 (#ET1702-34), smad3 (#ET1607-41), p-smad3 (#ET1609-41) were purchased from HUABIO (China); and tubulin (#AF1216) was purchased from Beyotime (China). Secondary HRP-conjugated antibodies (#S0101, #S0100) were purchased from Beyotime (China).

### Cell Viability Assay

RAW264.7 and MLE-12 were seeded in a 96-well plate at a density of 1 × 10^4^ cells/well. The cells were exposed to 12.5, 25, 50, 100, or 200 μg/ml of PM_2.5_ for 1, 12, 24, or 48 h. Cell viability was measured by using MTT (Sigma, Sigma Aldrich, St. Louis, MO) based on the protocol used in the previous study (15). Briefly, MTT (final concentration of 500 μg/ml) was incubated for 2 h at 37°C and then treated with 100 μl DMSO. Next, the absorbance was read in the microplate spectrophotometer (Synergy H1, BioTek, USA) at a wavelength of 492 nm. Data were expressed as the percentage of untreated cells.

### Isolation of sEV and TEM Observation

The sEV were isolated from conditioned medium or serum by ultracentrifugation according to the standard methods. Briefly, conditioned medium or serum was centrifuged at 300 g for 30 min to remove cells; 3,000 g for 30 min to eliminate cell debris, 10,000 g for 30 min to remove large particles, and then 120,000 g for 2 h to isolate sEV with a Type 70 Ti rotor (Beckman, Germany). The new supernatant was removed and the pellet sEV was resuspended in 100 μl sterile PBS. The sEV were stored at −80 °C before use. The transmission electron microscopy (Tecnai G2 F20 TWIN TMP 200 kV, FEI, USA) was applied to observe the ultrastructue of sEV.

### Nanoparticle Tracking Analysis

The isolated sEV was analyzed by nanoparticle tracking analysis (NTA) to determine the concentration and the size. sEV isolated from 1 ml of plasma or 10 ml cell supernatant were resuspended in 1 ml of PBS and then analyzed by NanoSight NS300 (Marvel, UK).

### sEV Labeling and Uptake

The sEV were labeled with PKH26 Fluorescent Cell Linker Kits for General Cell Membrane Labeling (Sigma, St. Louis, MO) based on the manufacturer's protocol. The labeled exosomes were incubated with HL-1 cells for 24 h. Cells were fixed with 4% paraformaldehyde (PFA) for 10 min, permeabilized with 0.1% Triton X-100 for 10 min, and then blocked by 3% BSA for 1 h at room temperature. The cell samples were incubated overnight with anti-tubulin primary antibody at 4°C, followed by incubation with secondary antibody labeled with Alexa Fluor 488 (#ZF-0512, ZSGB-BIO, China) for 1 h at room temperature. Nuclei were labeled with 4,6-diamido-2-phenylindole dihydrochloride (DAPI) (Beyotime, China). A Zeiss LSM 880 confocal microscope system was applied to capture images. A Zeiss LSM 880 confocal microscope system was applied to capture images.

### ELISA

The level of TGF-β, TNF-α, and IL-6 in sEV were analyzed with ELISA kits (CSB-E04726m, CSB-E04741m, CSB-E04639m, CUSABIO, China) based on the manufacturer's instructions. The data of signals were determined by a microplate spectrophotometer (Synergy H1, BioTek, USA). The concentration of cytokines was calculated according to the standard curve and OD value.

### Immunohistochemistry

Histopathology was performed as previously described. After perfusion with cold PBS, the heart sample was fixed with 4% paraformaldehyde (PFA) for 48 h, embedded with paraffin, and then cut into a heart section (5 μm). The tissue sections were stained with hematoxylin and eosin (H&E) stain and Masson's trichrome stain kit according to the standard techniques to evaluate the cardiac injury and collagen distribution of heart tissue. ImageJ software was used to determine the percentage of fibrotic area.

### Statistical Analysis

All data were expressed as means ± standard error of the means (SEM), and were analyzed with GraphPad Prism 9 software. If the data of two groups conformed to be normal distribution and homogeneity of variance, they were analyzed by a two-tailed *t*-test. Otherwise, they were analyzed by ANOVA (three or more groups) followed by Bonferroni's correction. For *in vitro* experiments, all the results were based on at least 3 independent experiments. A *p* < 0.05 was defined as statistical significance.

## Results

### PM_2.5_ Induced Cardiac Inflammation and Fibrosis

**Figure 1:**
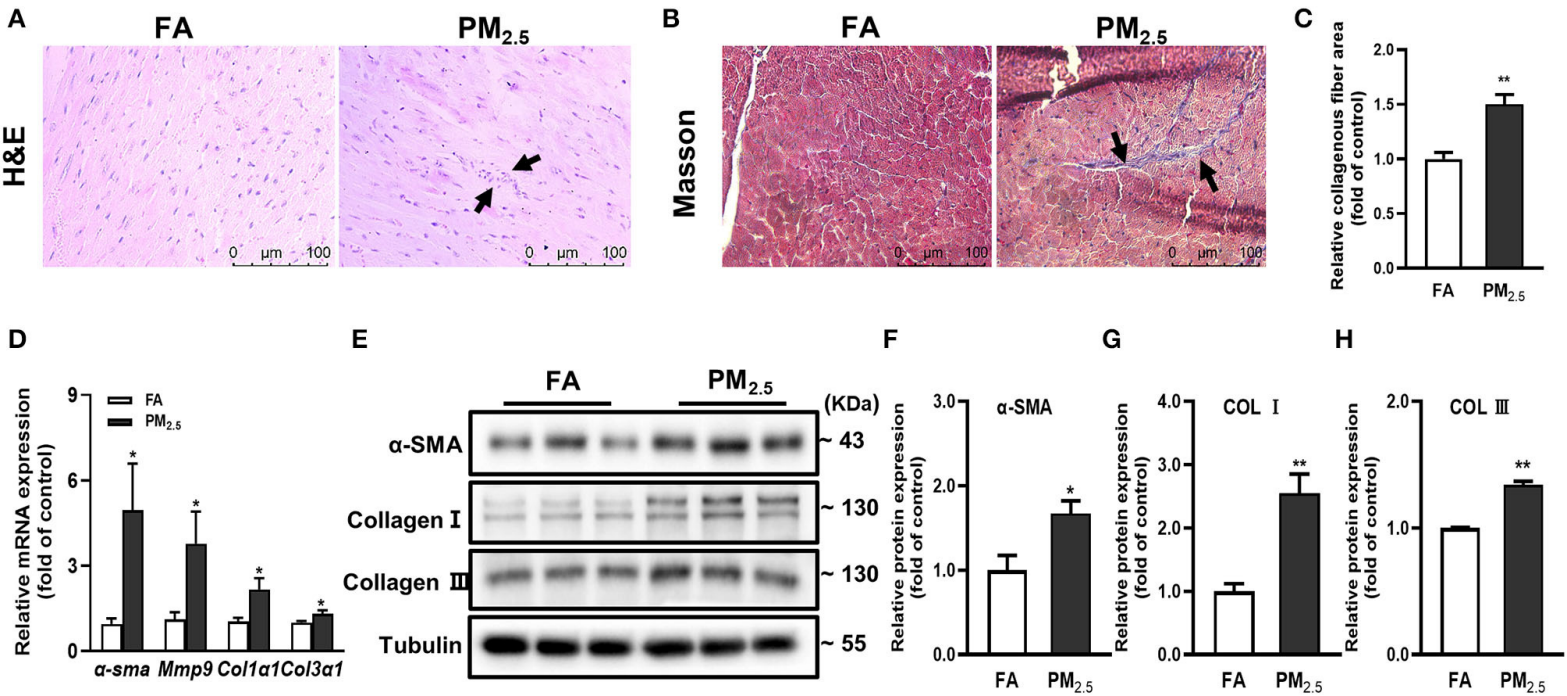
PM_2.5_ induced cardiac inflammation and fibrosis. **(A)** Representative images of hematoxylin-eosin (H&E) staining in heart issue, scale bars: 100 μm. The black arrow indicated the infiltration of inflammatory cells. **(B)** Representative images of Masson staining in heart issue, scale bars: 100 μm. **(C)** Quantitation of cardiac fibrosis area. **(D)** Relative mRNA expressions of α-SMA, mmp9, Col1α1, and Col3α1 in the hearts of mice were analyzed by qPCR. **(E–H)** Relative protein expressions of α-SMA, Col I and Col III in the hearts of mice were analyzed by Western blot. All the data were presented as mean ± SEM (*t*-test), *n* = 5, *indicates *p* < 0.05, **indicates *p* < 0.01.

To best mimic human exposure, mice were exposed to ambient airborne PM_2.5_ from Beijing Zhongguancun District for 4 months; more details were described in our previous study (14). To evaluate the effects of PM_2.5_ exposure on cardiac inflammation and fibrosis, we used hematoxylin and eosin (H&E) and Masson staining in heart tissues of mice. The results showed the obvious inflammatory cell infiltration (Figure 1A) and significantly increased myocardial collagen fibers (Figures 1B,C) in PM_2.5_ exposed mice. Besides, the mRNA and protein expressions of fibrosis markers, including α-smooth muscle actin (α-SMA), collagen I (Col I), and collagen III (Col III) were increased in the heart tissue of mice after PM_2.5_ exposure (Figures 1D–H). All the data proved that long-term PM_2.5_ exposure could induce cardiac inflammation and fibrosis.

### Serum sEV From PM_2.5_-Exposed Mice Increased the Level of Fibrosis-Related Proteins in Cardiomyocytes

**Figure 2:**
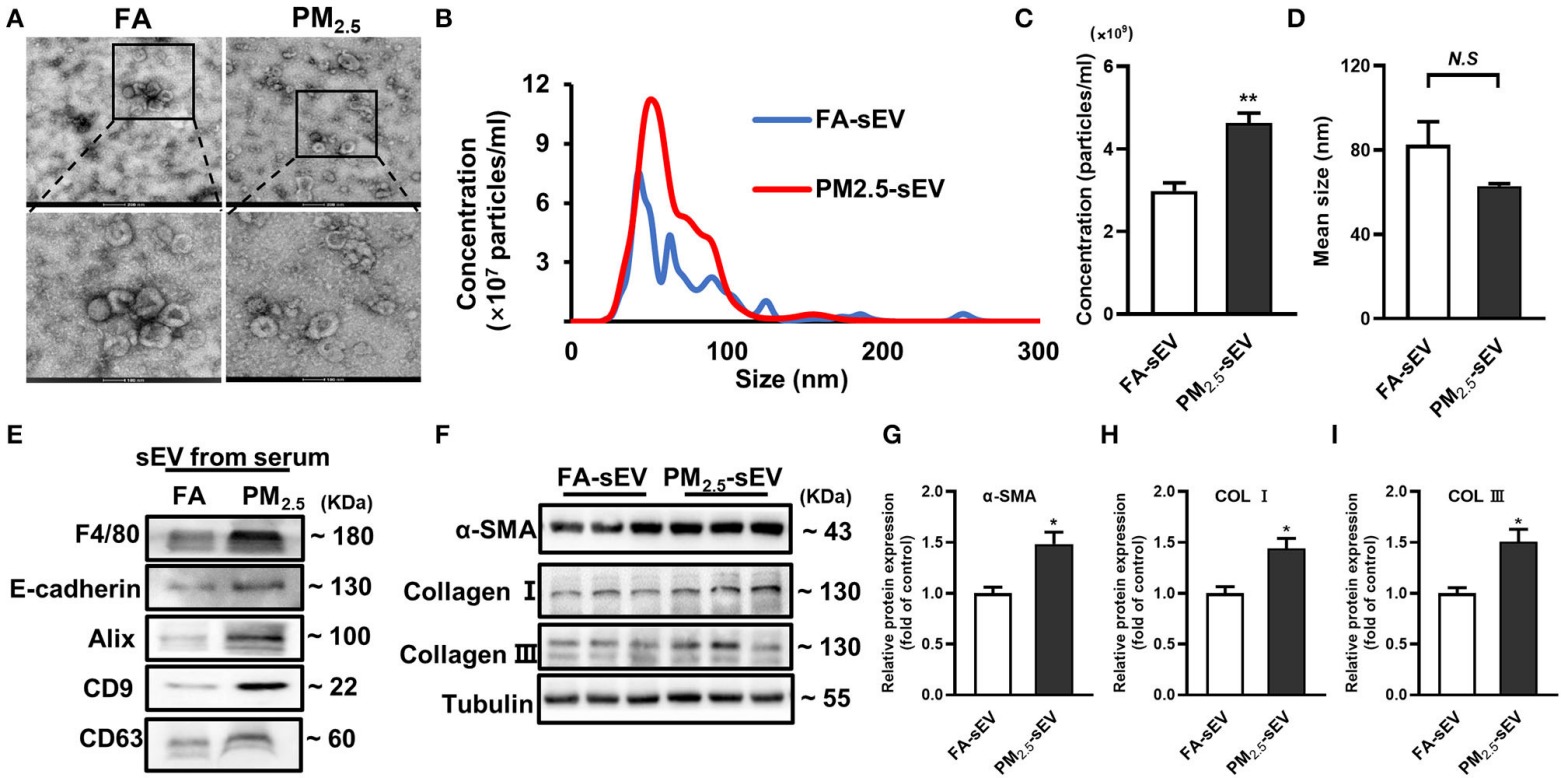
Serum sEV from PM_2.5_-exposed mice increased the level of fibrosis-related proteins in cardiomyocytes. **(A)** Representative TEM images of isolated serum sEV, scale bars: 500 nm, 100 nm. **(B)** Representative results of NTA demonstrated the concentration and distribution in FA-sEV and PM_2.5_-sEV. **(C)** The average concentration in FA-sEV and PM_2.5_-sEV. **(D)** Mean size in FA-sEV and PM_2.5_-sEV. **(E)** Representative images of Western blot in macrophage marker (F4/80), epithelial cell marker (E-cadherin), and sEV marker (Alix, CD9, and CD63) in FA-sEV and PM_2.5_-sEV. **(F–I)** Relative protein expressions of α-SMA, Col I, and Col III in HL-1 cells treated with sEV analyzed by Western blot (*n* = 4). All the data were presented as mean ± SEM (*t*-test), *n* = 5, *indicates *p* < 0.05, **indicates *p* < 0.01. N.S indicates no significance.

Our previous study has demonstrated that PM_2.5_ played a key role in overall heart failure progression by regulating lung oxidative stress, inflammation, and remodeling (16). To better understand how lung injury regulates cardiac inflammation and fibrosis, we isolated serum sEV from FA mice and PM_2.5_ exposed mice. Transmission electron microscopy (TEM) showed typical cup or saucer-like particles in two groups (Figure 2A). Nanoparticle tracking analysis (NTA) showed an obvious increase of concentration and no significant difference between FA-sEV and PM_2.5_-sEV (Figures 2B–D). Western blot confirmed that the isolated sEV were enriched with sEV marker proteins, including Alix, CD9, and CD63 (Figure 2E). However, we also detected two major lung cells surface markers, macrophage (F4/80) and epithelial cell (E-cadherin), in serum sEV. The notable increase of F4/80 implied that macrophage-sEV may be the critical factor of PM_2.5_-induced cardiac fibrosis.

To investigate whether sEV could regulate the progression of cardiac fibrosis, sEV were co-incubated with mouse cardiac muscle HL-1 cells for 24 h. PM_2.5_-sEV, but not FA-sEV, promoted the protein levels of α-SMA, Col I, and Col III in HL-1 cells (Figures 2F–I).

### sEV Released From PM_2.5_-Treated Macrophage Increased Collagen Expressions in Cardiomyocytes

**Figure 3:**
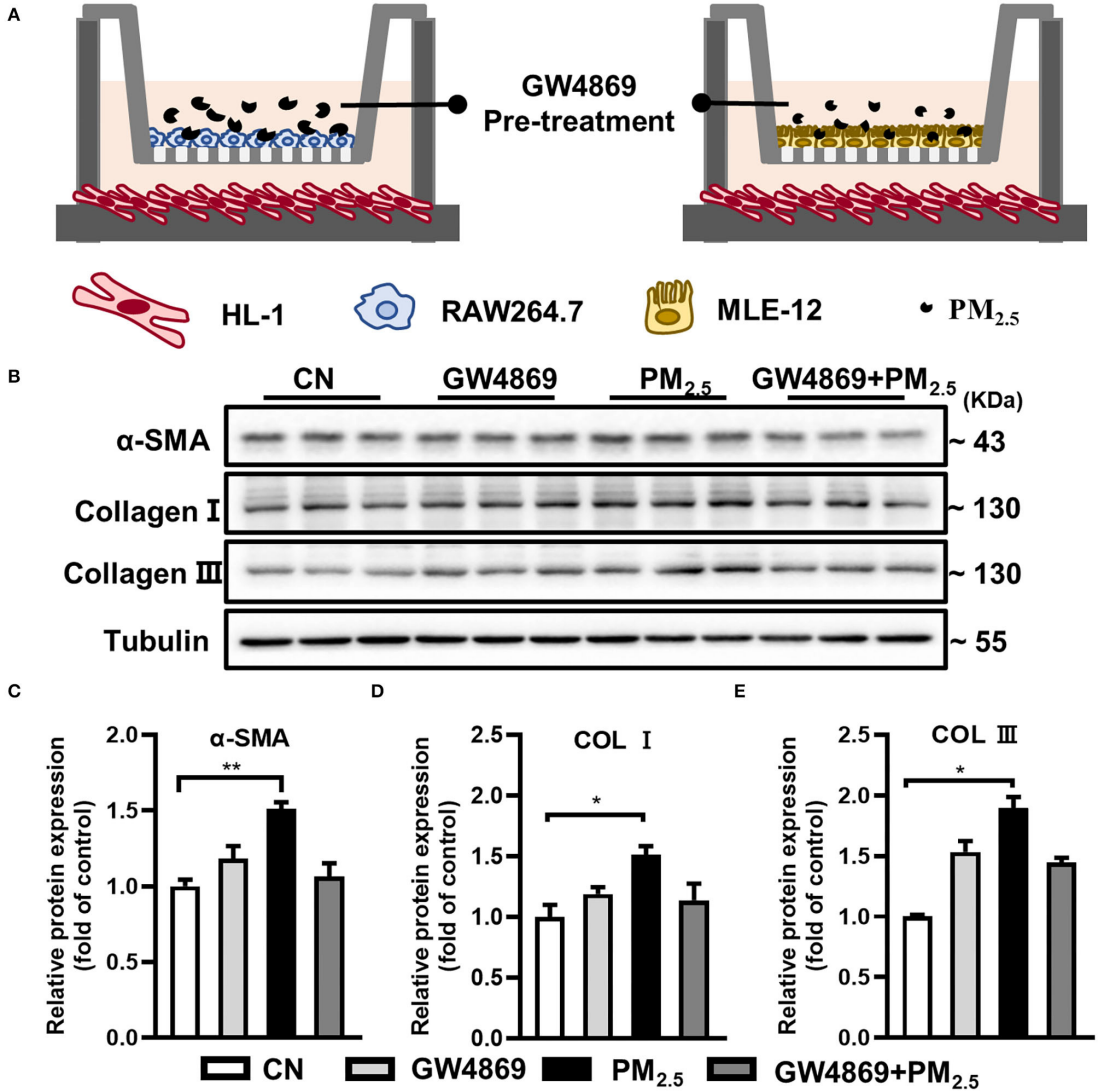
PM_2.5_-treated macrophage-sEV increased collagen expressions in cardiomyocytes. **(A)** HL-1 cells cocultured with RAW264.7 and MLE-12 in the presence or absence of PM_2.5_ and GW4869 in a transwell (0.4 μm) plate. **(B–E)** Cocultured with RAW264.7 and PM_2.5_ promoted the protein levels of α-SMA, Col I, and Col III in HL-1 cells through transwell system. All the data were presented as mean ± SEM (*t*-test, one-way ANOVA), *indicates *p* < 0.05, **indicates *p* < 0.01.

Due to the difficulties in distinguishing the major origin of sEV after PM_2.5_ exposure, a transwell co-culture system (0.4 μm)was used to confirm the crosstalk between PM_2.5_-exposure macrophages or epithelial cells and cardiomyocytes *via* sEV (Figure 3A). GW4869, a confirmed sEV secretion inhibitor, was added into the upper culture media for a 2-h-pretreatment in mouse macrophage cells (RAW264.7) and alveolar epithelial cells (MLE-12) (17). Then, we treated them with or without PM_2.5_. The results showed the obvious increase of α-SMA, Col I, and Col III in HL-1 cells cocultured with RAW264.7 and the tendency to decrease after pretreatment of GW4869 (Figures 3B–E). However, there was no significant change in HL-1 cocultured with MLE-12 (Supplementary Figure S1). All the data indicated that macrophage-derived sEV played a critical role in the regulation of cardiac fibrosis.

### Separation and Characterization of sEV From RAW264.7 and MLE-12 After PM_2.5_ Exposure

**Figure 4:**
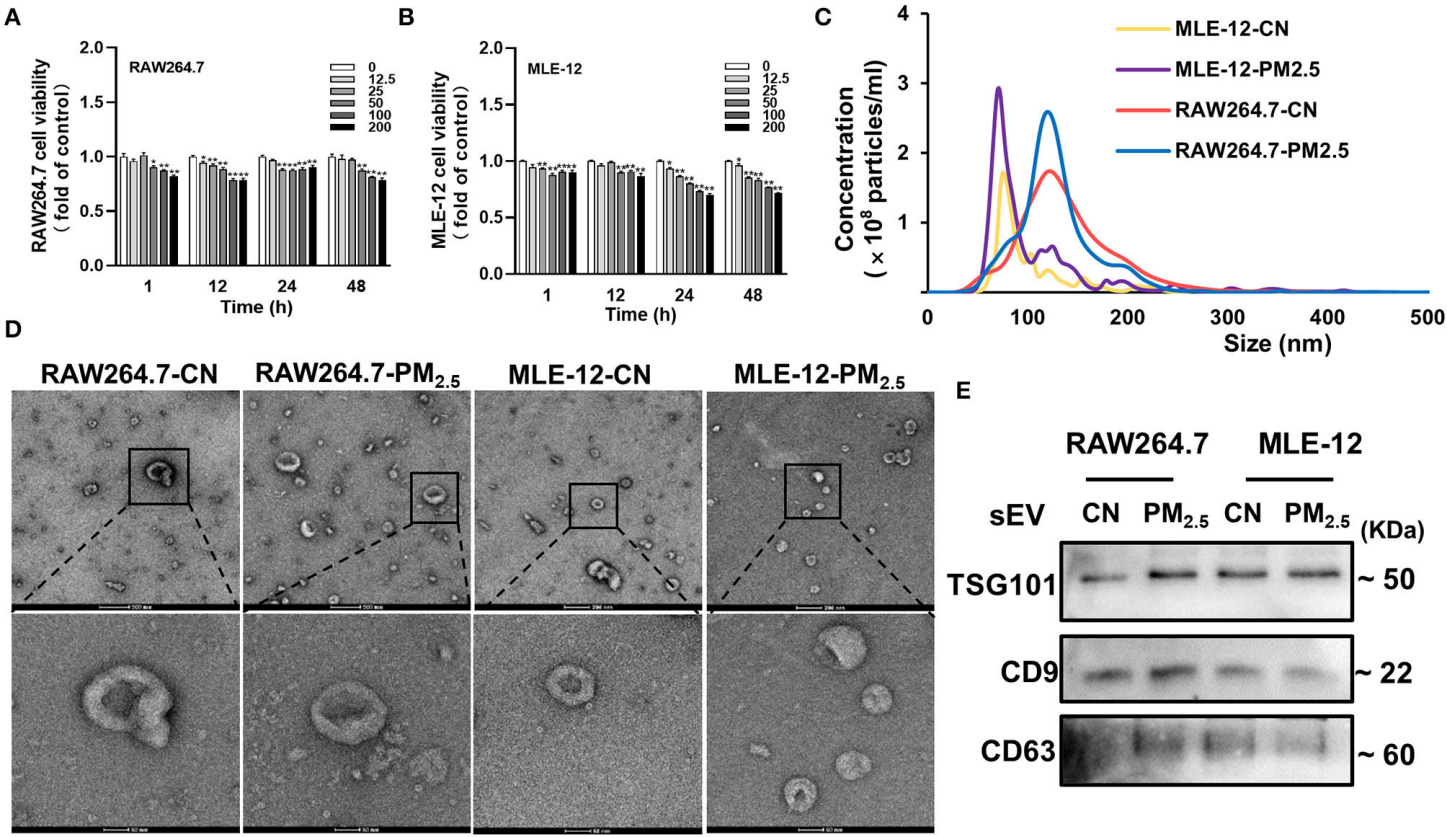
Separation and characterization of sEV from RAW264.7 and MLE-12 after PM_2.5_ exposure. **(A,B)** Cell viability analyzed by MTT assay. The RAW264.7 and MLE-12 were treated with various concentrations of PM_2.5_ for 1, 12, 24, and 48 h (*n* = 5). **(C)** The results of NTA demonstrated the concentration and distribution in CN-sEV and PM_2.5_-sEV. **(D)** Representative TEM images of isolated sEV, scale bars: 200 nm, 50 nm. **(E)** Representative images of Western blot in sEV marker Alix, CD9 and CD63 in CN-sEV and PM_2.5_-sEV. All the data were presented as mean ± SEM (*t*-test), *n* = 3–4, *indicates *p* < 0.05, **indicates *p* < 0.01.

To better explore the function of sEV from different cells in lung-mediated cardiac fibrosis induced by PM_2.5_ exposure, we isolated sEV from mouse macrophage cells (RAW264.7) and alveolar epithelial cells (MLE-12) with or without PM_2.5_-treated. We firstly applied MTT viability assay to test the effects of PM_2.5_ on RAW264.7 cells and MLE-12 cells. The cells were exposed to PM_2.5_ for 1, 12, 24 and 48 h at concentrations of 0 to 200 μg/mL and showed a concentration-dependent decrease (Figures 4A,B). A total of 50 μg/mL is chosen as our dosing concentration, of which 80–90% of the cells were viable compared with controls. NTA, TEM, and western blotting analyses were performed to identify the purity of sEV derived from RAW264.7 and MLE-12. NTA reflected the increase of sEV in both two types of cells after PM_2.5_ exposure (Figure 4C). TEM showed the representative images of sEV in two groups (Figure 4D). Western blotting showed the presence of sEV surface markers, including Alix, CD9, and CD63 (Figure 4E). All these data manifested that our purified nanoparticles were sEV.

### Release of Cytokines in sEV by RAW264.7 and MLE-12 After PM_2.5_ Exposure

**Figure 5:**
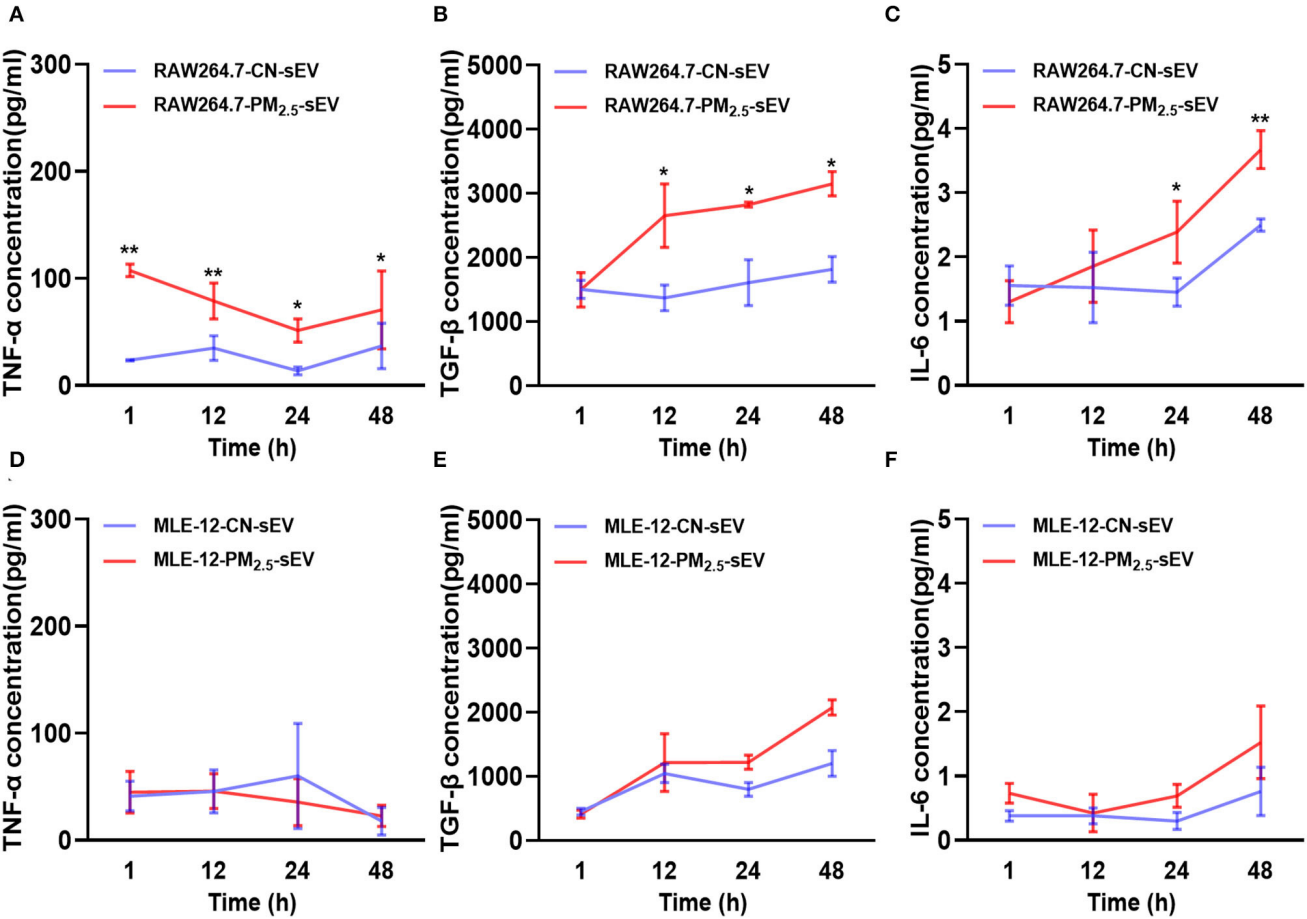
Release of key cytokines in sEV by RAW264.7 and MLE-12 after 1, 12, 24, 48 h of PM_2.5_ exposure. **(A–C)** ELISA for TNFα, TGF-β, and IL-6 in sEV from RAW264.7 cells after 1, 12, 24, and 48 h of PM_2.5_ exposure. **(D–F)** ELISA for TNFα, TGF-β, and IL-6 in sEV from MLE-12 cells at 1, 12, 24, and 48 h after PM_2.5_ exposure. All the data were presented as mean ± SEM (two-way ANOVA), *n* = 3. *indicates *p* < 0.05, **indicates *p* < 0.01.

Cytokines, including TNF-α, TGF-β, and IL-6, play remarkable roles in cardiac fibrosis, which combined with fibroblast surface receptors to activate fibrosis-related signal pathways (18, 19). We examined the release pattern of these cytokines in sEV from different cells after 1, 12, 24, and 48 h of PM_2.5_ exposure. We found that TNF-α increased rapidly in RAW264.7-derived sEV in the initial 1 h of PM_2.5_ exposure (Figure 5A). TNF-α from RAW264.7-sEV increased over exposure time and became steady till 48 h (Figure 5A). Besides, we observed a continuous growth of TGF-β content in RAW264.7-sEV (Figure 5B) and an increased IL-6 covering after PM_2.5_ exposure (Figure 5C). However, there was no significant difference in MLE-12-derived sEV after PM_2.5_ exposure, and the levels of cytokines in MLE-12-sEV were lower than RAW264.7-sEV (Figures 5D–F). Hence, PM_2.5_ exposure mainly altered TNF-α and TGF-β in macrophage-sEV, which may mediate the levels of fibrosis-associated proteins in cardiomyocytes.

### TGF-β-Containing sEV Induced Production of Collagen Through TGF-β-Smad2/3 Signaling Pathway in Cardiomyocytes

**Figure 6:**
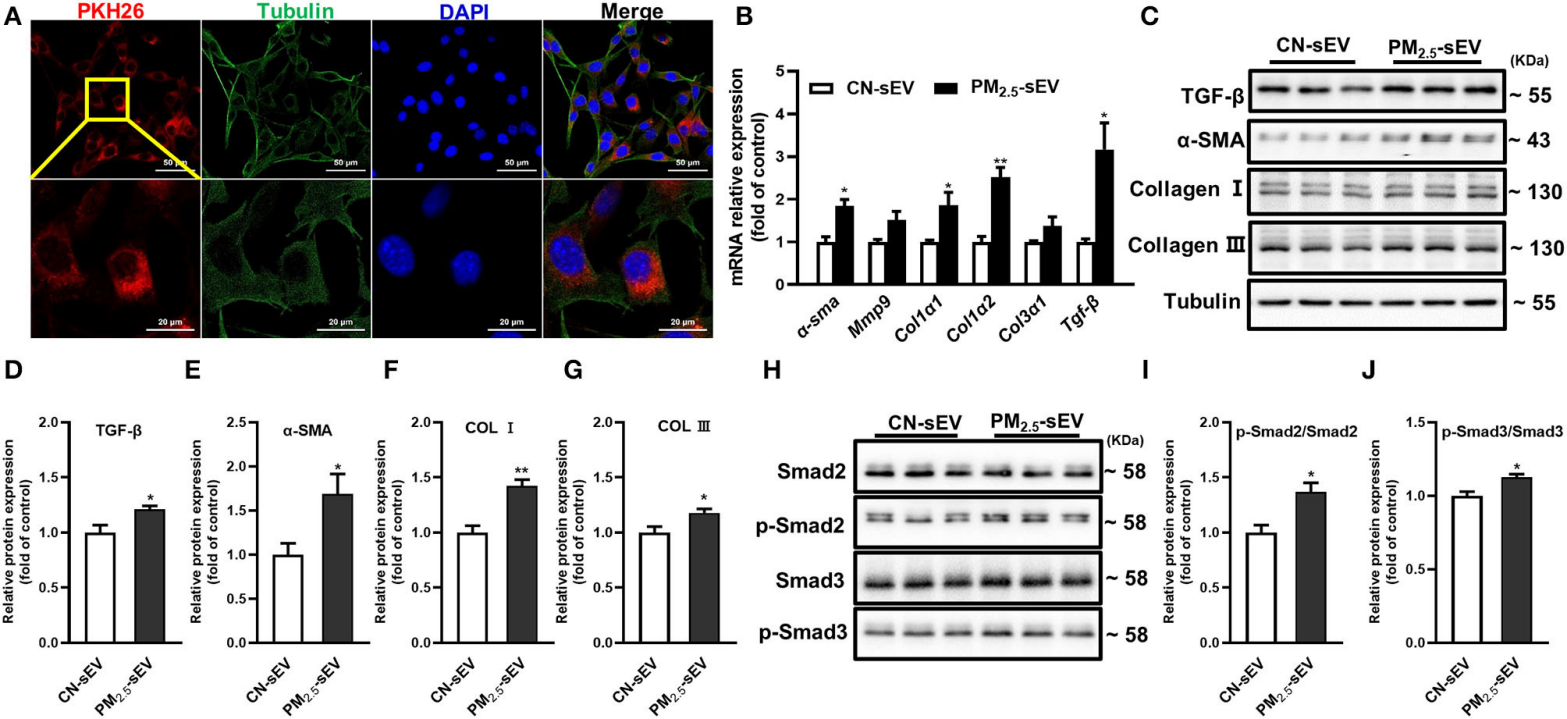
TGF-β-containing sEV-induced production of collagen through TGF-β-Smad2/3 signaling pathway in cardiomyocytes. **(A)** Representative images of HL-1 cells that were incubated with the RAW264.7-sEV of PKH26-labeled sEV (red), cytoskeleton was stained with tubulin (green) and nuclei with DAPI (blue). Scale bar: 50 μm, 20 μm. **(B)** Relative mRNA expressions of α-SMA, mmp9, Col1α1, Col1α2, Col3α1, and TGF-β in HL-1 after being treated with RAW264.7-sEV analyzed by qRT-PCR. **(C–G)** Relative protein expressions of GF-β, α-SMA, Col I, and Col III in HL-1 were analyzed by Western blot. **(H–J)** Quantification of normalized p-Smad2/Smad2, p-Smad3/Smad3 in HL-1. All the data were presented as mean ± SEM (*t*-test), *n* = 3–4, *indicates *p* < 0.05, **indicates *p* < 0.01.

To determine the regulation of TGF-β-containing sEV from macrophages on myocardial fibrosis process, firstly, we labeled sEV with red fluorescence PKH-26 and then cocultured them with HL-1 cells for 24 h. The intracellular uptake of labeled sEV was observed under a laser scanning confocal microscope (Figure 6A). Then, we detected the expression of the mRNA and protein expressions of TGF-β, α-SMA, Col I, and Col III in HL-1 cells. The results showed that PM_2.5_-induced RAW264.7-sEV could promote the mRNA and protein expressions of α-SMA, Col I and Col III (Figures 6B–G). Phosphorylation of Smad2/3 and total Smad2/3 in HL-1 cells were then analyzed. RAW264.7-sEV co-culture increased the protein expression of p-Smad2/Smad2 and p-Smad3/Smad3 (Figures 6H–J). Our data preliminary indicated that PM_2.5_ increased the expression TGF-β in macrophage–sEV, which activated the TGF-β-Smad2/3 signaling pathway in cardiomyocytes.

## Discussion

Our study showed that PM_2.5_ exposure modulated the intercellular communication between macrophages and cardiomyocytes, increasing TGF-β in macrophage sEV, which upregulated the levels of cardiomyocyte fibrosis-related proteins and aggravated myocardial fibrosis.

It is well accepted that air pollution is a significant cause of non-communicable diseases worldwide, and particulate matter is one of the main air pollutants (20). Although previous studies have demonstrated that particulate matter exposure activated the ROS/TGF-β1/Smad3 signaling pathway to induce myocardial fibrosis (21), our study explained that PM_2.5_ exposure not only increased cardiomyocyte and fibrosis levels by TGF-β, but also promotes this outcome through macrophage–sEV secretions.

Some studies focused on the role of sEV-induced chronic diseases by respiratory exposure, and it has been shown that exposure of the respiratory tract to ambient particulate matter promoted the secretions of sEV from different cellular sources and alter their composition (22). These sEV may remain in the lungs, or regulate systemic inflammatory responses through blood circulation. Plasma EV levels were significantly increased in short-term PM exposure populations and steel plant workers exposed to occupational PM, and the significantly altered miRNAs in sEV could regulate coagulation function, inflammatory response, and fibrin levels (23, 24). To expound whether PM_2.5_ exposure induced cardiac fibrosis through sEV, serum sEV were isolated from 4-month-exposure mice and cocultured with cardiomyocytes. The results reflected that serum sEV could induced myocardial fibrosis after PM_2.5_ exposure.

However, there are still some uncertainties about the potential sources of increased levels of serum sEV after exposure. Additional studies have reported that macrophage-derived sEV containing angiotensin II type 1 receptor played an important role in BLM-induced pulmonary fibrosis (25), and macrophage-derived sEV also activated the fibroblast in an endoplasmic reticulum stress-dependent manner to mediate silica-induced pulmonary fibrosis (26). In addition, sEV from alveolar epithelial cells activated alveolar macrophage in sepsis-induced acute lung injury (27), and cigarette smoke extract-treated lung epithelial Beas-2B-derived sEV could promote macrophage polarization (28). All these studies revealed the critical role of sEV from macrophages and lung epithelial cells in the regulation of lung microenvironment homeostasis. To investigate whether and how sEV from lung cells mediated myocardial fibrosis through blood circulation after PM_2.5_ exposure, we established sEV models of lung epithelial cells and macrophages *in vitro* to explore the regulation of sEV from different cells on cardiomyocyte fibrosis. We firstly used GW4869, a specifically selective inhibitor of N-SMase to reduce sEV release, which can successfully blocked sEV by pre-treatment of cells. The results reflected that PM_2.5_-induced RAW264.7-sEV increased α-SMA and collagen levels in HL-1 cells, while the similar results were not observed in MLE-12-sEV.

Afterward, we isolated sEV from RAW264.7 cells and MLE-12 cells. It is found that PM_2.5_ increased the level of sEV in two cells. A large amount studies have established that macrophages released a large amount of pro-fibrotic growth factors such as TGF-β, platelet-derived growth factors (PDGFs), and fibroblast growth factors (FGF) (29). Meanwhile, the pro-inflammatory cytokines IL-1β, IL-6, and TNF-α induced the transcription of IL-10, PDGF, or TGF-β to promote the fibrotic macrophage (M2c) phenotype (29). The growing evidences reflected that sEV transferred the cytokines to modulate the functions of recipient cells (30–32). According to previous studies, PM_2.5_ increased the levels of TGF-β1 in mice lung tissue and bronchoalveolar lavage fluid, suggesting that PM_2.5_ exposure may increase TGF-β1 and cause myocardial fibrosis (33). Thus, we detected the levels of TNF-α, TGF-β, and IL-6 from RAW264.7 cells and MLE-12 cells. Various cell exposure models at different times were designed to track the changes of cytokines in sEV. The rapid response of TNF-α in RAW264.7-derived sEV may promote macrophage polarization and pro-fibrotic factors transcriptions. However, with the increase of PM_2.5_ exposure time, the expression of TGF-β increased, which may be a major factor in the fibrosis process. In addition, the levels of cytokines in sEV from RAW264.7 cells significantly increased as compared with sEV from MLE-12 cells.

Finally, we found that macrophage-derived sEV upregulated the levels of TGF-β and fibrosis-associated proteins after PM_2.5_ exposure. TGF-β in cardiac tissue caused Smad2/Smad3 phosphorylation, which would affect various profibrotic gene expressions and stimulate cardiac fibrosis development (34). To gain a comprehensive understanding of collagen increase induced by macrophage-derived sEV, we measured the phosphorylation of Smad2/3 and total Smad2/3 in HL-1 cells. Our data revealed that PM_2.5_-induced TGF-β-containing sEV caused collagen deposition by activating TGF-β-Smad2/3 signaling pathway in cardiomyocytes.

To summarize, our study suggested that PM_2.5_-treated cell-to-cell communication between macrophages/lung epithelial cells and cardiomyocytes promotes cardiac fibrosis. More importantly and practically, this critical communication through sEV, which connects macrophages and cardiomyocytes, may provide new ideas for preventive treatment and facilitate the development of diagnosis and treatment for patients with PM_2.5_-associated CVD. However, there are still some deficiencies in our study, including that the regulatory role of other important components in sEV, such as miRNAs or proteins, on cardiac fibrosis has not been determined. In addition, the sEV inhibitor GW4869 can be used in mice to better confirm the regulation of sEV after PM_2.5_ exposure. These unanswered questions will provide a more comprehensive understanding of cardiovascular disease caused by PM_2.5_ exposure.

## Conclusion

In summary, our study put forward for the first time that the PM_2.5_ induced cardiac fibrosis by regulating the TGF-β-Smad3/2 signaling pathway *via* macrophage-derived sEV. This study provides a novel insight into the mechanism underlying PM_2.5_-induced cardiovascular diseases.

## Data Availability Statement

The original contributions presented in the study are included in the article/Supplementary Material, further inquiries can be directed to the corresponding authors.

## Ethics Statement

The animal study was reviewed and approved by University of Chinese Academy of Sciences Animal Care and Use Committee.

## Author Contributions

WD, FZ, and XH conceived, designed the research, and revised the manuscript. XH, MC, XC, and XY performed the experiments. XH analyzed the experiment results and edited the manuscript. All authors contributed to the article and approved the submitted version.

## Funding

This study was supported by the Major Program of National Natural Science Foundation of China (91643206).

## Conflict of Interest

The authors declare that the research was conducted in the absence of any commercial or financial relationships that could be construed as a potential conflict of interest.

## Publisher's Note

All claims expressed in this article are solely those of the authors and do not necessarily represent those of their affiliated organizations, or those of the publisher, the editors and the reviewers. Any product that may be evaluated in this article, or claim that may be made by its manufacturer, is not guaranteed or endorsed by the publisher.
