# Supplementary material for: TGF-β-Containing Small Extracellular Vesicles From PM2.5-Activated Macrophages Induces Cardiotoxicity
Source: Front Cardiovasc Med. 2022 Jul 8;9:917719. doi: 10.3389/fcvm.2022.917719 (PMC9304575; doi:10.3389/fcvm.2022.917719)
Supplement: Supplementary file 2 [file Data_Sheet_1.docx]

Supplementary material

**TGF-β-containing small extracellular vesicles from PM_2.5_-activated** **macrophages induces** **cardiotoxicity**

Xiaoqi Hu^1, 2^, Mo Chen^1, 2^, Xue Cao^1^, Xinyi Yuan^1^, Fang Zhang^1^*, Wenjun Ding^1,2^*

1 Laboratory of Environment and Health, College of Life Sciences, University of Chinese Academy of Sciences, Beijing 100049, China

2 Sino-Danish Center for Education and Research, Sino-Danish College, University of Chinese Academy of Sciences, Beijing, 100049, China

Running title: Cardiotoxicity induced by PM_2.5_ via small extracellular vesicles

*Corresponding author:

Wenjun Ding, PhD

College of Life Sciences, University of Chinese Academy of Sciences, No. 19A Yuquan Road, Beijing 100049, China

E-mail: dingwj@ucas.ac.cn

Fax: 86-10-69672641; Tel: 86-10-69672641

Fang Zhang, PhD

College of Life Sciences, University of Chinese Academy of Sciences, No. 19A Yuquan Road, Beijing 100049, China

E-mail: zhangfang@ucas.ac.cn

Fax: 86-10-88256460; Tel: 86-10-88256460

**Supplemental** **Figure 1**


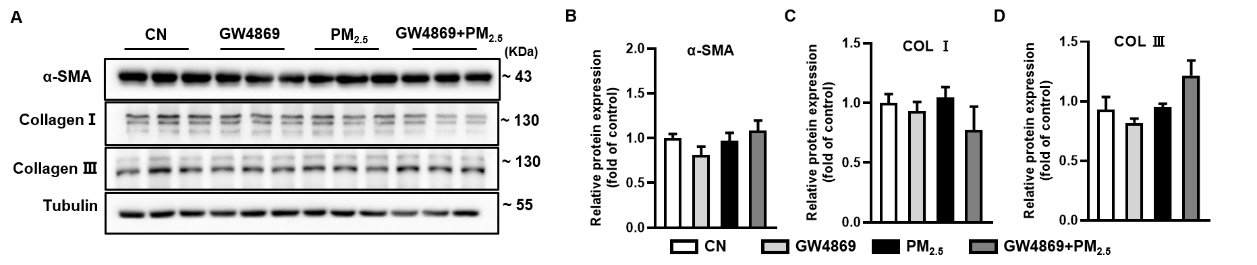


Figure S1. The protein levels of α-SMA, Col Ⅰ and Col Ⅲ in HL-1 cells cocultured with MLE-12 and PM_2.5_ through transwell system. All the data were presented as mean ± SEM (t-test, one-way ANOVA), n=3.
